# Age-related neurodegenerative disease associated pathways identified in retinal and vitreous proteome from human glaucoma eyes

**DOI:** 10.1038/s41598-017-12858-7

**Published:** 2017-10-04

**Authors:** Mehdi Mirzaei, Veer B. Gupta, Joel M. Chick, Todd M. Greco, Yunqi Wu, Nitin Chitranshi, Roshana Vander Wall, Eugene Hone, Liting Deng, Yogita Dheer, Mojdeh Abbasi, Mahdie Rezaeian, Nady Braidy, Yuyi You, Ghasem Hosseini Salekdeh, Paul A. Haynes, Mark P. Molloy, Ralph Martins, Ileana M. Cristea, Steven P. Gygi, Stuart L. Graham, Vivek K. Gupta

**Affiliations:** 1 Department of Chemistry and Biomolecular Sciences, Macquarie University, Sydney, NSW Australia; 2 School of Medical Sciences, Edith Cowan University, Joondalup, WA Australia; 3 Department of Cell Biology, Harvard Medical School, Boston, Massachusetts USA; 4 Department of Molecular Biology, Princeton University, Princeton, New Jersey USA; 5 Faculty of Medicine and Health Sciences, Macquarie University, Sydney, NSW Australia; 6 Centre for Healthy Brain Ageing, School of Psychiatry, University of New South Wales, Sydney, Australia; 7 Save Sight Institute, Sydney University, Sydney, NSW Australia; 8 Department of Molecular Systems Biology, Cell Science Research Center, Royan, Institute for Stem Cell Biology and Technology, ACECR, Tehran, Iran; 9 Australian Proteome Analysis Facility, Macquarie University, Sydney, NSW Australia

## Abstract

Glaucoma is a chronic disease that shares many similarities with other neurodegenerative disorders of the central nervous system. This study was designed to evaluate the association between glaucoma and other neurodegenerative disorders by investigating glaucoma-associated protein changes in the retina and vitreous humour. The multiplexed Tandem Mass Tag based proteomics (TMT-MS3) was carried out on retinal tissue and vitreous humour fluid collected from glaucoma patients and age-matched controls followed by functional pathway and protein network interaction analysis. About 5000 proteins were quantified from retinal tissue and vitreous fluid of glaucoma and control eyes. Of the differentially regulated proteins, 122 were found linked with pathophysiology of Alzheimer’s disease (AD). Pathway analyses of differentially regulated proteins indicate defects in mitochondrial oxidative phosphorylation machinery. The classical complement pathway associated proteins were activated in the glaucoma samples suggesting an innate inflammatory response. The majority of common differentially regulated proteins in both tissues were members of functional protein networks associated brain changes in AD and other chronic degenerative conditions. Identification of previously reported and novel pathways in glaucoma that overlap with other CNS neurodegenerative disorders promises to provide renewed understanding of the aetiology and pathogenesis of age related neurodegenerative diseases.

## Introduction

Glaucoma represents one of the major causes of irreversible blindness in the elderly. This complex degenerative disorder is characterised by progressive and selective loss of retinal ganglion cells (RGCs)^1^ in the inner retina. The axons of RGCs form the core component of the optic nerve, relaying visual information detected by the retina to the brain. Therefore, a loss of structural and functional integrity of RGSs plays a major role in the development and progression of visual deficits reported in common optic neuropathies and particularly in glaucoma^2^. While several risk factors have been identified that are associated with premature loss of RGCs, high intraocular pressure is currently the most significant risk factor in glaucoma^3^. The molecular mechanism(s) that result in RGC dysfunction in various optic neuropathies however remain ill-defined. Greater understanding of the underlying neurodegenerative processes is crucial for the development of effective therapeutic strategies for glaucoma.

It is increasingly recognized that glaucoma and AD show overlap of several molecular pathological features including β-amyloid accumulation in the retina^4–6^. AD is characterized by the presence of extracellular plaques containing abnormal amyloid β (Aβ) aggregates as well as intracellular neurofibrillary tangles composed of hyper-phosphorylated tau, a microtubule-associated protein localised in axons. Clinically, patients exhibit a progressive decline in memory, cognition and learning, which are accompanied by visual and ocular manifestations, similar to glaucoma^5,7^. These pathological features may explain the impaired contrast sensitivity and motion perception often reported in AD patients. There is evidence for a positive association of glaucoma in patients with AD and the involvement of Aβ and hyper-phosphorylated tau in ocular degeneration in glaucoma patients^8,9^.

We have previously demonstrated that AD is associated with ocular deficits including inner retinal thinning and impaired retinal electrophysiological response in animal models of AD^10^. Similar anatomical and functional deficits predominantly localised to the inner retina have also been reported in clinical studies in AD subjects^11^. However, the molecular bases of these links remain obscure mainly due to the limited availability of data concerning biochemical and “omics” related retinal changes either in glaucoma and/or AD. Importantly, the lack of sufficient information about which molecular changes are involved in either glaucoma or AD pathology precludes either an early and accurate disease diagnosis, or the development of critical target-based therapeutic development.

In this study, we investigated the molecular basis of glaucoma pathogenesis by taking a systems level perspective of the retina and vitreous proteome using unbiased quantitative proteomics approaches. Large-scale proteome profiling of human post-mortem tissues were conducted, comparing protein abundances in vitreous and retinal tissues from ‘control’ and glaucoma eyes. This study provides significant mechanistic advancements into the common molecular pathways implicated in the pathogenesis of glaucoma and other neurological disorders with a particular focus on AD as a representative neurodegenerative condition.

## Methods

### Human eye samples, retina and vitreous extraction

Frozen human post-mortem eye tissues (20 donors) from open angle glaucoma (9 male, 1 female) and age matched control (8 male, 2 female) subjects who had consented to the use of their tissues for research purposes were obtained from the Sydney Eye Bank, NSW (Lions NSW Eye Bank), Australia. Human ethics approval was obtained from the Macquarie University Human Research Ethics committee. Research was carried out in accordance with the principles outlined in the declaration of Helsinki. Both the retinal and vitreous tissues were examined (average ages: control 64.5 ± 10, n = 10 and glaucoma: 71.5 ± 8.5, n = 10, respectively). Tissues were obtained within 6 hours after death. A history of glaucoma had been obtained from the donors’ medical records. None of the subjects had a known history of AD, Parkinson’s disease, ocular surgery, macular degeneration or other ocular disorders affecting visual function. Frozen eyes were allowed to thaw and washed with PBS; retina, optic nerve head and vitreous tissues were carefully removed from the eyecups without the retinal pigment epithelium (RPE) layer contamination under the surgical microscope (Carl Zeiss, Oberkochen, Germany).

### Preparation of protein samples

Retina and vitreous tissues were lysed in lysis buffer (20 mM HEPES, pH 7.4, 1% Triton X-100, 1 mM EDTA) containing 10 μg/ml aprotinin, 10 μM leupeptin, 1 mM PMSF and 1 mM NaVO_3_, 100 mM NaF, 1 mM Na_2_MoO_4_ and 10 mM Na_4_P_2_O_7_ and sonicated using a probe sonicator (3 pulses/15 s/50 Hz with 20 s between each pulse). Insoluble materials were removed by centrifugation at 15,000 g for 10 min at 4 °C. Extracted proteins were reduced with 5 mM DTT for 15 minutes at room temperature, and then alkylated with 10 mM iodoacetamide for 30 minutes in the dark at room at 4 °C. The alkylation reaction was then quenched with addition of 5 mM DTT for 15 minutes in the dark.

In order to remove the interfering detergents and contaminants, proteins were precipitated using the chloroform methanol precipitation protocol^12^. The protein pellet was resuspended in 200 μl 8 M Urea in 50 mM Tris (pH 8.8). Protein concentration was determined by BCA assay kit (Pierce, Rockford, USA) using bovine serum albumin (BSA) as a standard. Dual digestion was carried out on 150ug protein, initially with Lys-C (Wako, Japan) at a 1:100 enzyme: protein ratio overnight at room temperature, followed by Trypsin (Promega, Madison, WI) at a 1:100 enzyme: protein ratio for at least 4 hours at 37 °C. Samples were then acidified with TFA to a final concentration of 1% (pH 2 to 3) and desalted using SDB-RPS (3M- Empore) Stage Tips^13^.

### TMT labelling

**Figure 1:**
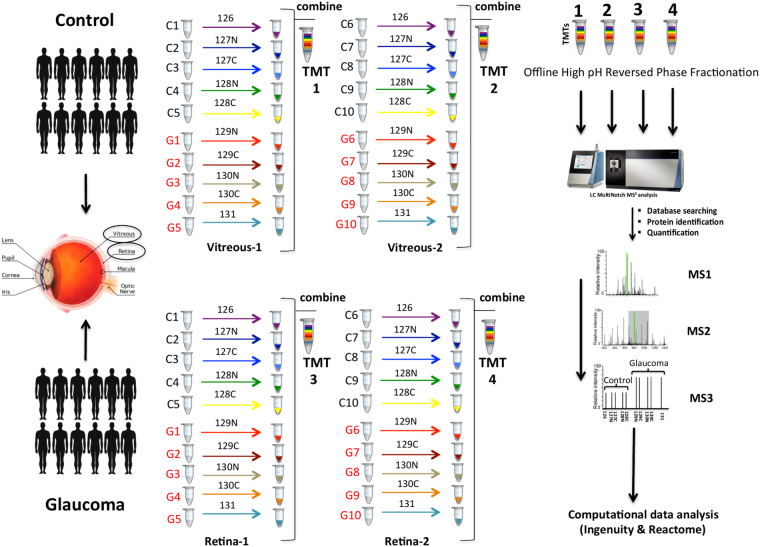
Experimental design and TMT labelling workflow of the experiment. Human retinal and vitreous tissues were extracted from postmortem eyes of control (age: control 64.5 ± 10, n = 10) and glaucoma subjects (age: 71.5 ± 8.5, n = 10). Extracted proteins form 40 samples subjected to reduction, alkylation and subsequent digestion with Trypsin and Lys-C. Extracted peptides were quantified and labeled in a 10 plex TMT reaction. Four TMT experiments were carried out to accommodate all the biological replicates. Briefly, 2 sets of 5 control and 5 glaucoma replicates of retina tissue were used in TMT 1 and 2 experiments, and the same design was used for vitreous samples in TMT3 and TMT4 experiments. Labelled samples within each TMT experiment were pooled together, then were fractionated by basic, reversed-phase isocratic step elution using reverse phase spin columns, and analyzed by LC-ESI-MS/MS on ThermoFisher Orbitrap Fusion mass spectrometer (SPS-MS3 method). Functional pathway and protein network data analysis was performed using Ingenuity and Reactome pathway analysis.

To accommodate the 40 biological samples (10 control and 10 glaucoma for retina and vitreous tissues each), four separate 10plex TMT experiments were carried out (two TMT experiments for retina and two for vitreous tissues). A detailed experimental design and TMT workflow is illustrated in Fig. 1. Briefly, dried peptides were resuspended in 200 mM HEPES (pH 8.2) and peptide concentration was measured using the MicroBCA protein assay kit (Thermo Scientific- Rockford, IL). 70 μg of peptides from each sample were subjected to TMT labelling with 0.2 mg of 20 μl reagent per labelling reaction. Labelling was carried out at RT for an hour with occasional vortexing. To quench any remaining TMT reagent and reverse tyrosine labelling, 8 μl of 5% hydroxylamine was added to each sample, this was followed by vortexing and incubation for 15 mins at RT. For each of the four 10plex experiments the 10 labelled samples were combined, and then dried down by vacuum centrifugation. Each of the TMT experiments was fractionated by basic, reversed-phase isocratic step elution using reverse phase spin columns (Pierce). Samples were loaded onto the reverse phase cartridges and elution was performed using 12 fractionation steps with the following acetonitrile concentrations in 10 mM ammonium bicarbonate (5, 10, 12.5, 15, 17.5, 20, 22.5, 25, 27.5, 30, 40, 80% ACN). These fractions were then pooled into 6 subsets (Fraction 1–7, 2–8, 3–9, 4–10, 5–11, 6–12). Each fraction was dried and then desalted using SDB-RPS (3M- Empore) Stage Tips.

### Liquid chromatography electrospray ionization tandem mass spectrometry (LC-ESI-MS/MS)

Fractionated peptide samples were reconstituted in 30 μl of 0.1% formic acid and 10 μl of samples were analysed using an Orbitrap Fusion Tribrid-MS (Thermo Scientific, USA) equipped with an ultra-high pressure liquid chromatography system (Proxeon). Peptides were separated for 3-hours on a reverse phase column with a gradient of 6–30% acetonitrile in 0.125% formic acid at a flow rate of ~400 nl/min. In each data collection cycle, one full MS scan (400–1400 m/z) was acquired in the Orbitrap (120,000 resolutions at 400 m/*z* and an AGC of 2 × 10^5^). MS3 was performed using HCD with 55% collision energy and reporter ion detection in the Orbitrap with an AGC of 150,000 ions, a resolution of 60,000 and a maximum ion accumulation time of 150 ms. Peptide fragmentation and collection of reporter ion spectra were performed using the synchronous precursor selection (SPS-MS3 method)^14^. In this method, first MS2 analysis was conducted using CID fragmentation on the top 10 most intense ions with following settings: normalized collision energy of 35%, AGC 4 × 10^3^, isolation window 0.5 Da, maximum ion accumulation time 150 ms with 40 seconds of dynamic exclusion. Following each MS2 scan, for the MS3 analyses, precursor isolation was performed using a 2.5 Da window and fragmented in the ion trap using CID as above, except with an AGC setting of 8,000. Multiple fragment ions (SPS ions) were co-isolated and further fragmented by HCD at normalized collision energy (NCE) of 37.5%. Selection of fragment ions was based on the previous MS2 scan and the MS2-MS3 was conducted using recently described sequential precursor selection (SPS) methodology^14^.

### Database searching/quantification and statistical analysis

In-house software tools were used to convert RAW file to the mzxml format^15^. Correction of erroneous charge state and monoisotopic m/z values were performed using method detailed in Huttlin *et al*.^16^. Sequence assignment of MS/MS spectra were made with the Sequest algorithm^17^ using an indexed human Uniprot database prepared with forward and reversed sequences concatenated as per the target-decoy strategy^18^. Data searches were conducted using cysteine carbamidomethylation and TMT on the peptide N-termini and lysine residues as static modifications, oxidation of methionine as a dynamic modification, precursor ion tolerance of 20 ppm and a fragment ion tolerance of 0.8 Da (for CID). Sequest matches were filtered using linear discriminant analysis to a false discovery rate (FDR) of 1% at the peptide level based on matches to reversed sequences, as previously reported^18^. The final peptide-level FDR fell well below 1% (~0.2% peptide level). A reductionist model was used for assignment of peptides to protein matches, where all peptides were explained using the least number of proteins. Protein rankings were generated by multiplying peptide probabilities and the dataset was finally filtered to 1% protein FDR.

Quantitation of peptides using TMT reporter ions was performed as previously published^15,19^. Briefly, a 0.003 Th window centred on the theoretical m/z value of each reporter ion was recorded for each of the 10 reporter ions, and the intensity of the signal closest to the theoretical m/z value was recorded. TMT signals were also corrected for isotope impurities as per manufacturer’s documentation. Peptides were only considered quantifiable if the total signal-to-noise (S/N) for all channels was >200 and a precursor isolation specificity of >0.75. Within each TMT experiment, reporter intensities values were normalized by summing the values across all peptides within each channel and then each channel was corrected so that each channel had the same summed value. Protein quantitation was performed by summing the normalized S/N values for all peptides assigned to a given protein. The protein quantitations from the two TMT experiments for each respective sample type (vitreous and retina) were aggregated into a single report. The proteins were regarded as differentially expressed based on a two sample t-test p-value (p ≤ 0.05) and a fold change threshold (≥1.3 for up-regulation or ≤0.76 for down-regulation). The reproducibility of all four TMT experiments output was evaluated by further statistical analysis such as overall data quality, un-supervised analyses such as clustering and PCA analysis, all implemented using our in-house ‘TMTPrepPro’ software^20^.

#### Electrochemiluminescence (ECL) assay

Selected proteins identified by quantitative mass spectrometry as being differentially abundant in glaucoma *versus* control samples and also previously reported to be altered in AD were analysed using a highly sensitive electrochemiluminescence (ECL) based MSD platform. Serum amyloid A (SAA), C-reactive protein (CRP), soluble vascular adhesion molecule 1 (sVCAM1), soluble intercellular adhesion molecule 1 (sICAM1), alpha-2-macroglobulin (A2M), beta-2 microglobulin (B2M), Factor VII (FVII), adiponectin, clusterin and Tenascin (TNC) were quantified in human retinal and vitreous samples from control and glaucoma subjects. Aliquots from tissue lysates were prepared according to the manufacturer’s instructions for each panel of assays. Assay plates were washed and blocked using the supplied buffers. Samples and standards were then loaded in duplicate into each assay plate, sealed and incubated at room temperature for 2 hours. Plates were washed using PBST (pH 7.4), then secondary detection antibodies added and plates were re-sealed and incubated again for 1 hour. Plates were finally washed 3 times with PBST, read solution added according to the assay instructions and read using a Sector Imager 1200 plate reader (MSD, Maryland, USA). The supplied software was used to determine standard curve and sample concentration, according to 5 parameter logistic curve-fitting techniques.

#### Western blotting

Western blot analyses were carried out on selected proteins (CLU, VTN, CRYBB2 and CRYBB3) to confirm the proteomic data. 25 μg of proteins from each sample was separated by 4–12% SDS-PAGE and transferred to PVDF membrane. Membranes were blocked by Tris-buffered saline containing 5% milk for 1 hour, and primary antibodies added at a final concentration of 1–2 μg/ml. After incubation with the primary antibody overnight at 4 °C, blots were washed with Tris-buffered saline and then incubated with anti-IgG antibody linked to horseradish peroxidase for 2 hours. Signals indicative of protein levels were detected using an enhanced chemiluminescent substrate (Clarity Western ECL Substrate, Bio Rad) and Bio-Rad ChemiDoc MP detection system and band intensities quantified in the linear range of detection.

#### Bioinformatics and functional pathway analysis

Pathways enrichment analysis was carried out on differentially expressed proteins using Ingenuity Pathway software (Ingenuity® Systems, www.ingenuity.com), Reactome Functional Interaction (FI) network (http://www.reactome.org/) and ClueGO. For Ingenuity, the gene identifiers and their fold change value (glaucoma vs. control) for each tissue were uploaded separately to the software. Identified proteins were correlated to corresponding gene using Ingenuity Pathway Knowledge Base (IPKB). Significant interaction networks (p < 0.05) and molecular and cellular functions were identified based on known protein–protein interactions in the published literature (knowledge base). Networks were “named” on the most common functional group(s) present. Canonical pathway analysis allowed us to identify the function-specific genes significantly present within the networks. For specific functional protein ontologies that were differentially regulated, the list of differentially expressed proteins for both retina and vitreous were analysed by the Reactome Functional Interaction (FI) network Cytoscape plug-in. All relevant data are available in the paper and its Supporting Information files.

## Results

### Differential protein regulation in glaucoma eye tissues quantified by multiplexed proteomics

We identified 4765 proteins from retinal tissue, and 4987 proteins from vitreous humour, which were quantified by multiple peptides at an initial protein FDR of less than 1% (see Supplementary data S1 for retina and Supplementary data S2 for vitreous). A series of descriptive statistical analyses were performed to confirm the reproducibility of the data. The overall distribution of median-normalized and log-transformed protein abundances were visualised in density and box plots (Supplementary Figure 1). Within each tissue (retina and vitreous), the boxplots showed similar median and 95% confidence intervals. Moreover, comparison of density plots for each individual biological sample showed similar and highly overlapping patterns, with no major asymmetric bias, satisfying the normality assumption for further analysis. Overall, these statistical metrics confirm the similarity in overall protein abundance distributions of individual biological replicates, and demonstrate the reproducibility of measurements within sample groups.

**Figure 2:**
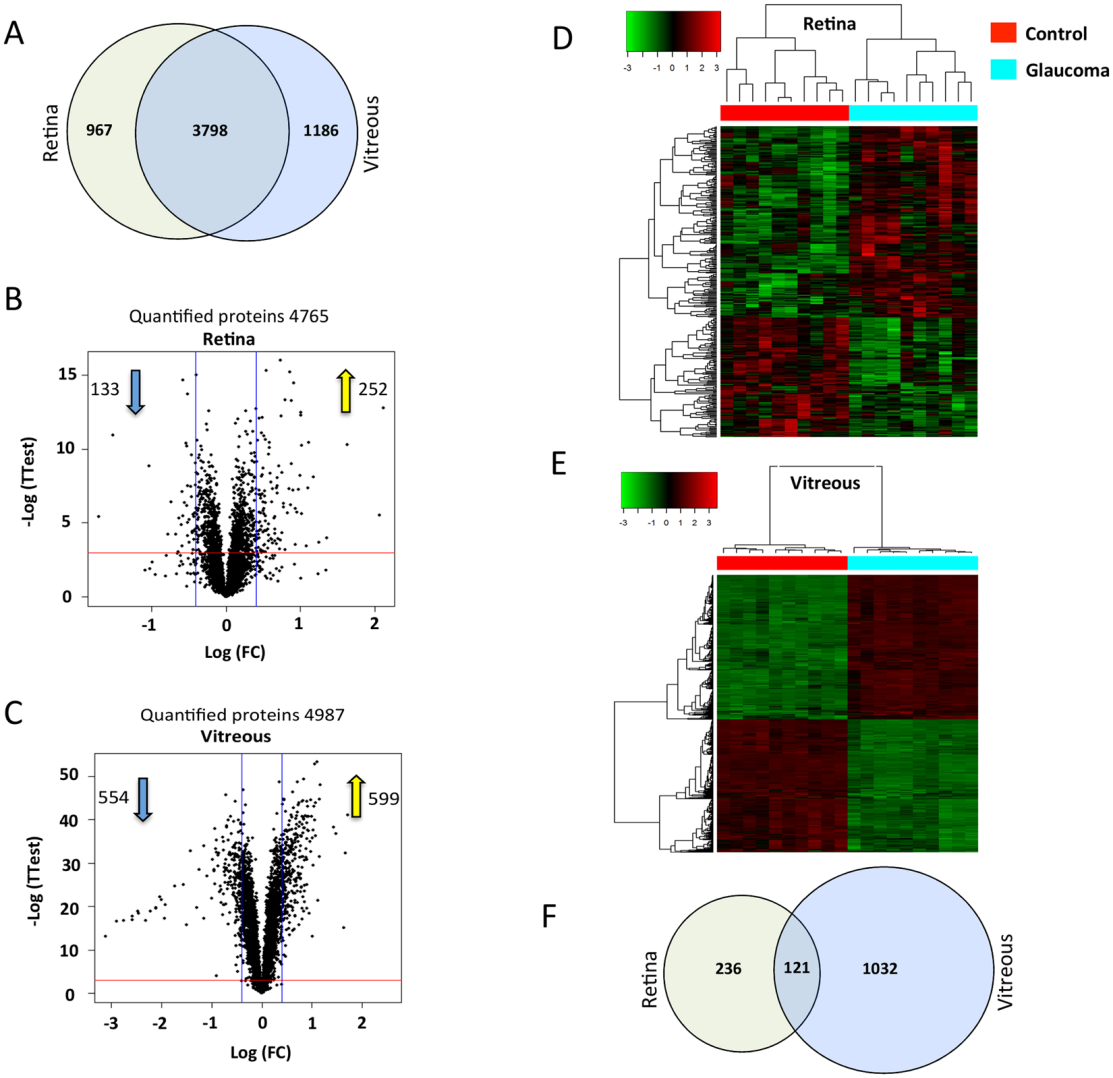
Results of proteomics analysis and quality control measurements. (**A**) Venn diagram indicating the overlap between the proteins identified and quantified from vitreous and retinal tissues (1% FDR). (**B,C**) Volcano plots demonstrating the dual thresholds for differentially regulated proteins. Each data point represents a single quantified protein. The x-axis represents log fold change in abundance (glaucoma/control). Vertical blue lines indicate 1.3 and 0.77 ratio. The −log (p-value) is plotted on the y-axis. Proteins above the red horizontal line indicate significance ≤ 0.05. Proteins within the upper and outer quadrants meet both the fold change and p-value cut-off, and are therefore considered as differentially regulated. (**D,E**) Heatmaps (hierarchical clustering) of the log-transformed ratios of differentially expressed proteins (glaucoma vs. control) in retina and vitreous. Column colours indicate treatment type. Red and green color-coding indicate relative increase or decrease in protein abundance, respectively. (**F**) Venn diagram representing the overlap between the differentially regulated proteins quantified in vitreous and retinal tissues (glaucoma vs. control, p-value ≤ 0.05, ≥ 1.3-fold or ≤ 0.77-fold.

To identify differentially expressed proteins, we used a combination of statistical and empirical thresholds to ensure a high confidence in the reported protein abundance differences. Quantified proteins were retained if their abundances in the glaucoma versus control patients were (1) statistically significant (p-value ≤ 0.05) using a student t-test and (2) differed by at least ± 30%. This two-step differential analysis of glaucoma versus control for retina tissue yielded 252 up regulated proteins (p-value ≤ 0.05 and ≥ 1.3-fold change) and 133 down regulated (p-value ≤ 0.05 and ≤ 0.77-fold change (Fig. 2B). In comparison, a greater proportion of differentially expressed proteins were identified in vitreous, with 554 and 599 proteins exhibiting increased and decreased abundance, respectively (Fig. 2C). Hierarchical clustering analysis of proteins with differential abundance illustrated the overall consistency of the up and down regulation within respective control and glaucoma cohorts (Fig. 2D and E). However, when comparing differential proteins between the vitreous and retinal tissues, we observed only moderate overlap (Fig. 2F). Given the overall retinal and vitreous proteomes were qualitatively similar (see Fig. 2A), the differences in these proteins suggested tissue specific dysregulation in glaucoma.It is possible that the cellular pathways dysregulated in glaucoma or their regulation is distinct in these tissues.

### Cellular pathway and protein network analysis reveals functionally coordinated protein abundance changes

To explore these possibilities in greater depth and gain more detailed information about the molecular mechanisms and biological processes that are altered in glaucoma, we performed cellular pathway enrichment and functional protein network analyses on the differentially expressed proteins of vitreous (1130 proteins) and retinal tissues (355 proteins).

**Figure 3:**
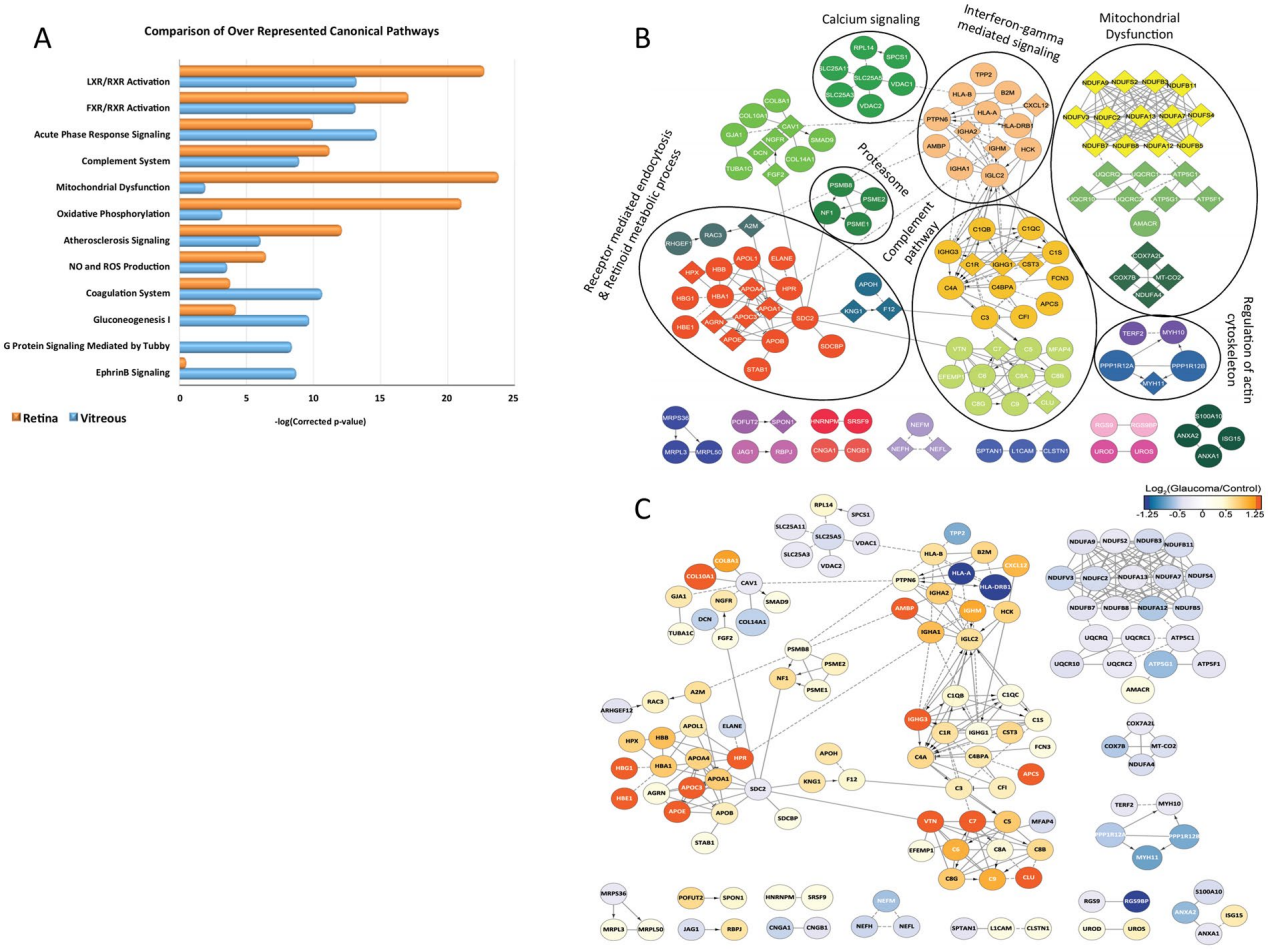
Results of functional protein interaction network and pathway analysis. (**A**) Comparison of the top 10 canonical pathways enriched from IPA analysis of differentially regulated proteins (glaucoma vs. control) from vitreous and retina tissues. The significance of functional enrichment is plotted on the y-axis as the −log (p-value). (**B**) Retinal Reactome functional interaction networks analyzed by the Reactome FI Cytoscape plugin. Of 355 retinal differentially expressed proteins, 136 proteins had at least one other known functional connection. Network nodes are labeled with gene symbols. The Reactome plugin was used to assign functional clusters, which were color-coded and labelled with representative broad functions. (**C**) Reactome network in (**B**) color-coded by log_2_ (glaucoma/control) relative abundance quantified from the TMT analysis.

Analysis of the differentially regulated proteins using Ingenuity Pathway Analysis (IPA) revealed that the top canonical pathways associated with immune and inflammatory pathways (e.g. LXR/RXR activation, FXR/RXR activation, acute phase response, and the complement system) were similarly over-represented in the vitreous and retina (Fig. 3A). In contrast, mitochondria-related pathways, such as, oxidative phosphorylation, NO/ROS production, and mitochondrial dysfunction were more significantly enriched in the retina (Fig. 3A, *orange bars*), while the coagulation system, gluconeogenesis I, and G protein and EphrinB signalling pathways were enriched more significantly in the vitreous (Fig. 3A, *blue bars*).

To visualize the functional connectivity among these differential proteins, we assembled together retinal and vitreous protein networks using the Reactome analysis tool. A high degree of connectivity, with 136 of the 357 differential proteins in the retina and 568 of the 1153 differential proteins in vitreous, forming interconnected networks (Fig. 3B and Supplementary Figure S2) was identified. High network connectivity for the retinal proteins involved in mitochondrial dysfunction, complement pathway, and receptor-mediated endocytosis and retinoid metabolic processes was observed (Fig. 3B). Within clusters of functionally connected proteins, the relative abundance in glaucoma and control tissues was similar (Fig. 3C). For example, 13 subunits of the NADH dehydrogenase: ubiquinone oxidoreductase complex I (NDUFA9, NDUFS2, NDUFB3, NDUFB11, etc), four subunits of cytochrome b-c1 complex III (UQCRQ, UQCRC2, UQCR10, UQCRC2), and four subunits of complex IV (COX7B, COX7A2L, NDUFA4, MT-CO2) in the mitochondrial inner membrane were all down regulated, while majority of the functional cluster containing components of the complement signalling cascade (C1QB, C1QC, C1R, etc.) were up regulated. Since the total number of differential proteins in vitreous was higher compared to that of the retina, significantly higher number of network clusters were identified, representing a wider array of cellular pathways in vitreous than in the retina. These included complement and coagulation pathways, PI3K-AKT signalling, cholesterol metabolism, apoptotic process, glycolysis, proteasome, RNA processing, mitochondrial ribosomal machinery, glutathione metabolism, oxidative phosphorylation, calcium signalling, synaptic vesicle transport, PPAR signalling, ion trans-membrane transport and receptor-mediated endocytosis (Supplementary Figure S2). As with the retinal functional protein network (Fig. 3B), functional clusters were largely co-ordinately regulated, including an upregulated cluster with RNA processing functions, and a ten protein cluster associated with glutathione metabolism, which was down regulated (Supplementary Figure 3). Overall, the combination of canonical IPA pathway and Reactome-based protein network analysis enabled statistical evaluation and visualization of the most significantly over-represented cellular processes that were impacted in retina and vitreous glaucoma tissues.

### Functional pathway analysis identifies association with Alzheimer’s disease markers in the retina and vitreous

**Figure 4:**
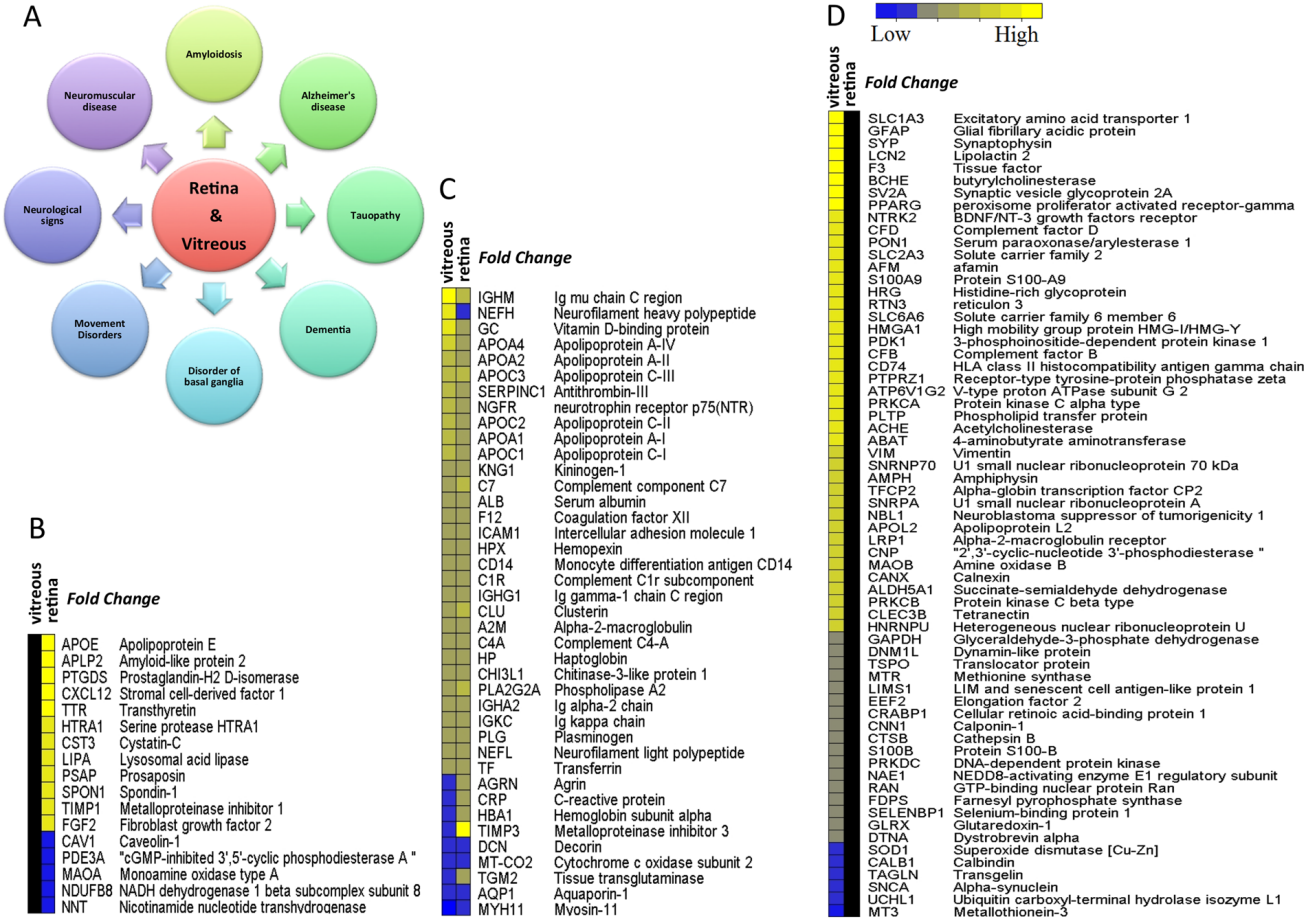
Top disease related biological functions and the list of 122 AD associated markers. (**A**) Representation of the top disease-related biological functions, which were commonly enriched in retina and vitreous. (**B**–**D**) Heatmaps of the log-transformed abundance ratios (glaucoma vs. control) quantified in retina and vitreous datasets for the 122 AD-associated proteins. Black columns indicate the corresponding protein either was not changed or detected in the dataset, yellow and blue represent up and down regulation, respectively. Relative abundances of (**B**) 16 AD-associated proteins identified only in the retina, (**C**) 40 common AD-associated proteins identified both in the retina and vitreous, and (**D**) 65 AD-associated proteins identified exclusively in the vitreous.

IPA analysis was performed to investigate the top disease-related biological functions commonly associated with the differential proteins in the retina and vitreous. This analysis highlighted networks associated with neuropathological diseases such as amyloidosis, tauopathy, and dementia (Fig. 4A). Specifically, out of 1510 differentially expressed proteins from retina and vitreous tissues analysed by IPA, 122 were assigned specifically to dementia and AD categories, of which 40 proteins overlapped between the retina and vitreous (Fig. 4C). While 15 proteins were uniquely expressed in the retina (Fig. 4B), the remaining 65 proteins were exclusive to vitreous tissue (Fig. 4D). Majority of these proteins were directly associated with the top IPA canonical pathways (Fig. 3A), including mitochondrial dysfunction, oxidative phosphorylation, acute phase response signalling, complement signalling, and the coagulation cascade. Based on the bioinformatics evidence linking glaucoma cellular pathology associated with neurodegenerative disorders of the brain, in the following sections, we discuss several examples of pathways and functional protein classes that have common pathophysiology with glaucoma and other CNS disorders particularly AD.

### Down-regulation of the mitochondrial electron transport chain proteins

Mitochondria play a central role in regulating neuronal cell survival and its impairment is associated with cell death since it governs both energy metabolism and apoptosis^21^. The energy required for cell growth, development and differentiation is provided by mitochondria in the form of ATP produced *via* oxidative phosphorylation (OXPHOS)^22^. The mitochondrial OXPHOS machinery comprises five key enzyme complexes consisting of 80 proteins, of which 13 are coded by the mitochondrial DNA. The mRNAs for these proteins are translated on mitochondrial ribosomes. The malfunction of ribosomal machinery is associated with decreasing the capacity for protein synthesis, followed by a significant reduction in ribosomal RNA and tRNA levels and subsequently up-regulation of RNA oxidation^23^. This dysregulation in protein synthesis lead to OXPHOS malfunction affecting all complexes, except the complex II. The electron transport chain is major source of reactive oxygen species (ROS) that accelerates oxidative damage followed by lipid peroxidation that eventually leads to OXPHOS damage and culminates in cell death due to energy restriction^24^. These processes are considered converging features of neurodegenerative diseases, and have been implicated in both glaucoma^25^ and AD^26–28^.

**Figure 5:**
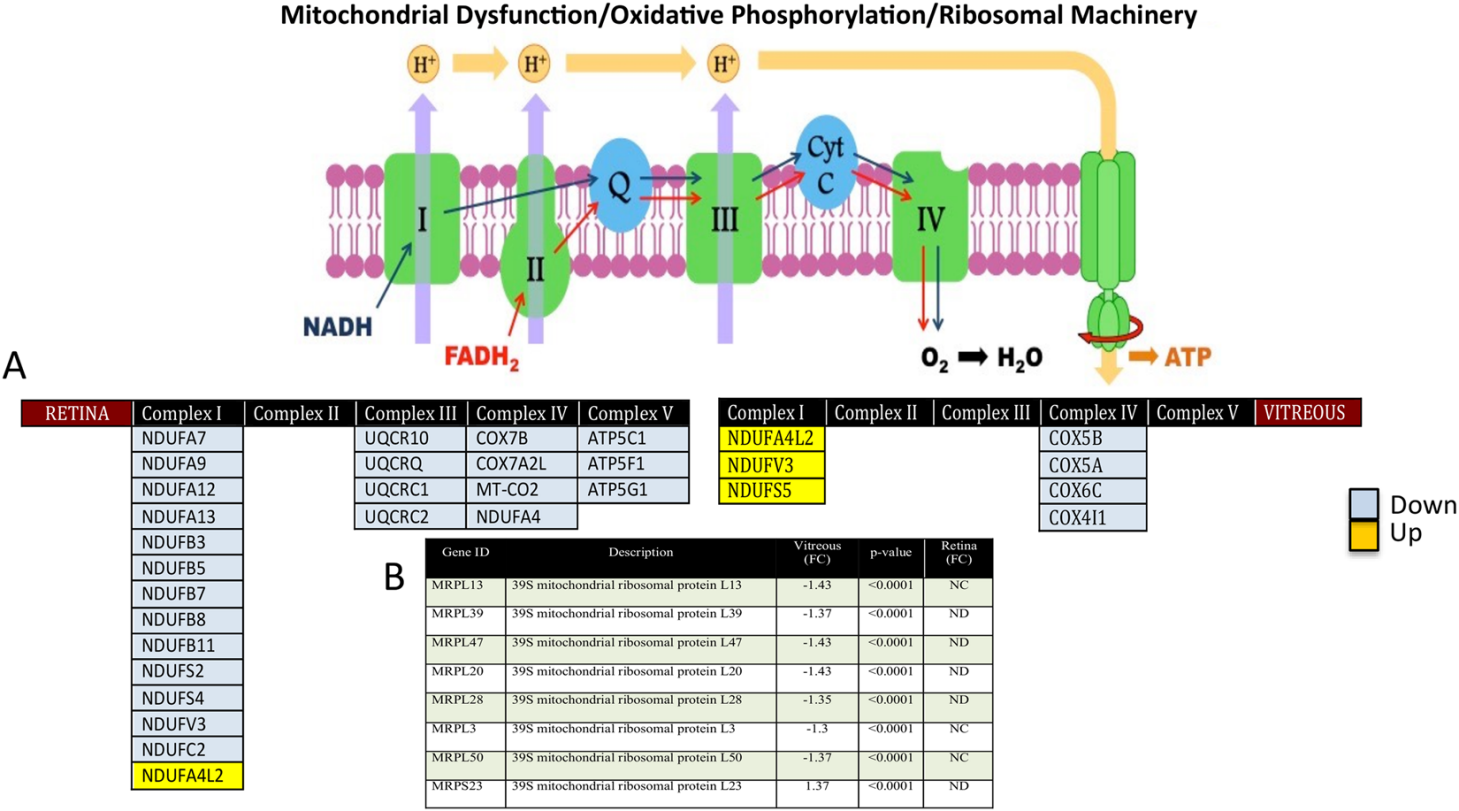
Down regulation of ETC proteins and mitochondrial ribosomal proteins in glaucoma. (**A**) A schematic diagram representing the overall down regulation of proteins associated with the electron transport chain (ETC) complexes of the mitochondria in glaucoma condition. (**B**) A table representing the down regulation of 7 mitochondrial ribosomal proteins in vitreous, which were not detected (ND) or not significantly altered (NC) in the retina (p- value ≤ 0.05).

In our dataset, we identified 32 retinal and 16 vitreous proteins associated with mitochondrial dysfunction and oxidative phosphorylation, plus seven proteins linked to the mitochondrial ribosomal machinery. Of the 32 retinal proteins, 24 were identified as down regulated, and belonged to outer and inner membranes and ETC complexes I–V, including 13 subunits of complex I (NADH: ubiquinone oxidoreductase), 4 subunits of complex III (Ubiquinol cytochrome c-oxidoreductase), 4 subunits of complex IV (cytochrome c oxidase complex) and 3 subunits of complex V (ATP synthase). In contrast, in the vitreous, proteins in complex III and V were not affected, and 3 subunits of complex I (NDUFV3, NDUFA4L2 and NDUFS5) were up regulated although these were down regulated in the retinal tissue. Similarly, four subunits of complex IV (COX5B, COX5A, COX6C and COX4I1) were downregulated in the vitreous but not the retina (Fig. 5A). All seven mitochondrial ribosomal proteins including MRPL13, MRPL39, MRPL47, MRPL20, MRPL28, MRPL3, MRPL50, MRPS23 were exclusively down regulated in vitreous, but were either not detected or did not change significantly in the retinal tissue (Fig. 5B).

### Activation of classical complement and coagulation cascades

**Figure 6:**
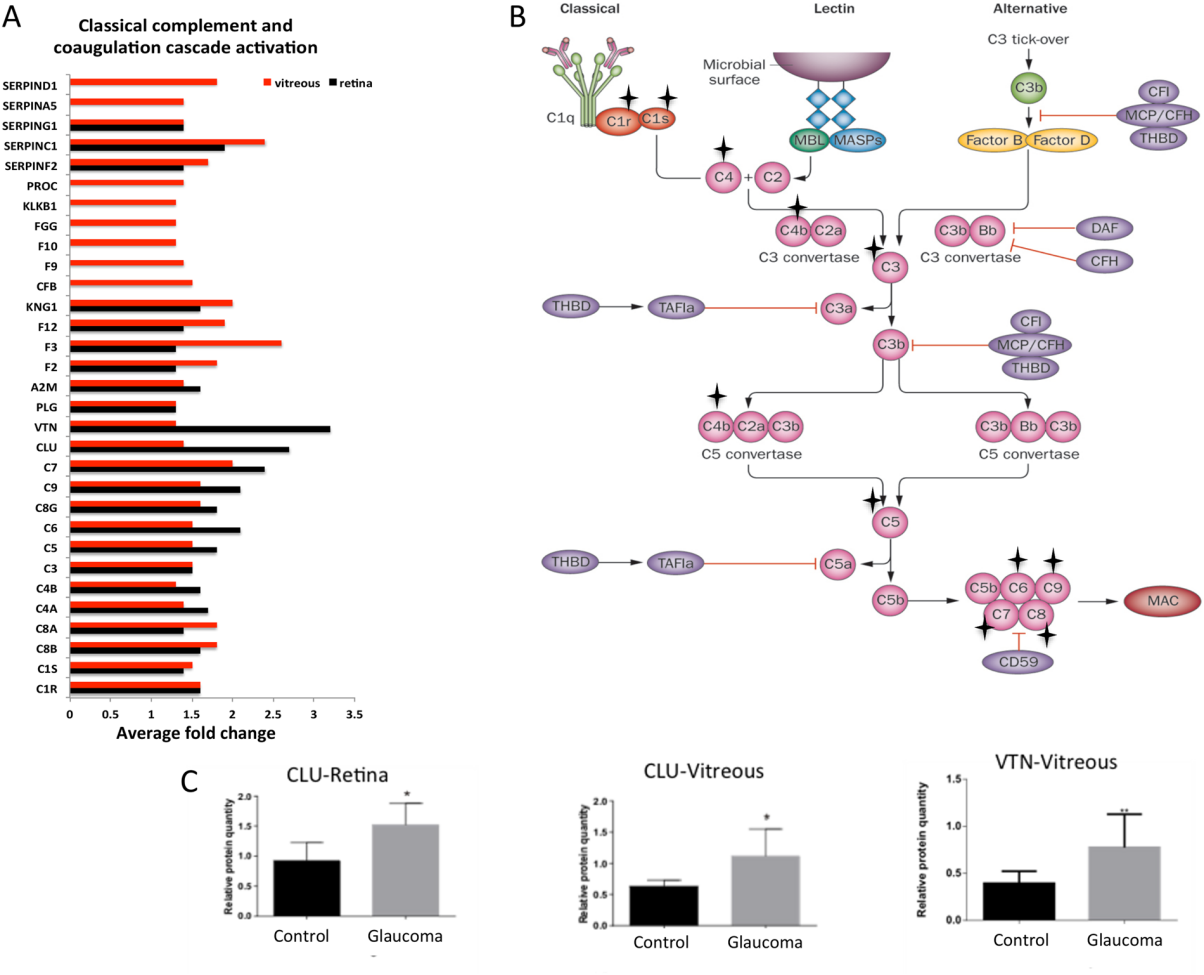
Activation of complement (classical) and coagulation cascades. (**A**) A bar graph representing the comparison of the relative abundance of proteins involved in the complement and coagulation cascade. The x-axis represent the average fold changes (p-value ≤ 0.05 and ≥1.3-fold change, n = 10). (**B**) A schematic diagram showing the details of the three pathways activating the complement cascade (classical, lectin and alternative), of which our quantitative proteomics data supports the activation of the classical pathway in both retina and vitreous. ★, indicates differentially regulated proteins that are part of the classical pathway (**C**) Western blotting analysis measuring the protein level of CLU (retina and vitreous, n = 10) and VTN in vitreous (n = 10) of glaucoma and control samples. GAPDH was used as the loading control. The bar graphs indicate average densitometry measurements (ImageJ software) (n = 10, average ± sd, *p-value < 0.05). Black bars represent control, while the light grey bars represent glaucoma. The copyright of the basic complement pathway image belongs to Noris, Mescia, & Remuzzi, Nat. Rev Neprh. 2012.

In agreement with previous studies in glaucoma^29–31^ and AD^32–34^, the activation of classical complement pathway in glaucoma patient samples was also observed in this study. We quantified the majority of key proteins associated with classical complement pathway, such as C1q, C1s, C1r, C4a, C4b, C3, C5, C6, C7, C8a, C8b, C8g and C9, as up-regulated in glaucoma condition for both vitreous and retinal tissues when compared to the controls. Supporting these findings, two membrane attack complex (MAC) assembly endogenous inhibitors, vitronectin and clusterin, were also found to be upregulated in glaucoma (Fig. 6A,B). The expression of these inhibitory proteins was further validated using western blotting (Fig. 6C). The coagulation cascade exhibited a similar expression pattern, where the majority of proteins involved such as F2, F3, A2M, PLG, KNG1, CFB, F9, F10, FGG, KLB1, PROC were up-regulated in glaucoma. However, higher expression of these proteins was observed in vitreous samples compared to that of the retina. Additionally, F9, F10, FGG, KLKB1 and PROC were exclusive to the vitreous tissue (Fig. 6A).

### Induction of cholesterol metabolism and transport proteins

**Figure 7:**
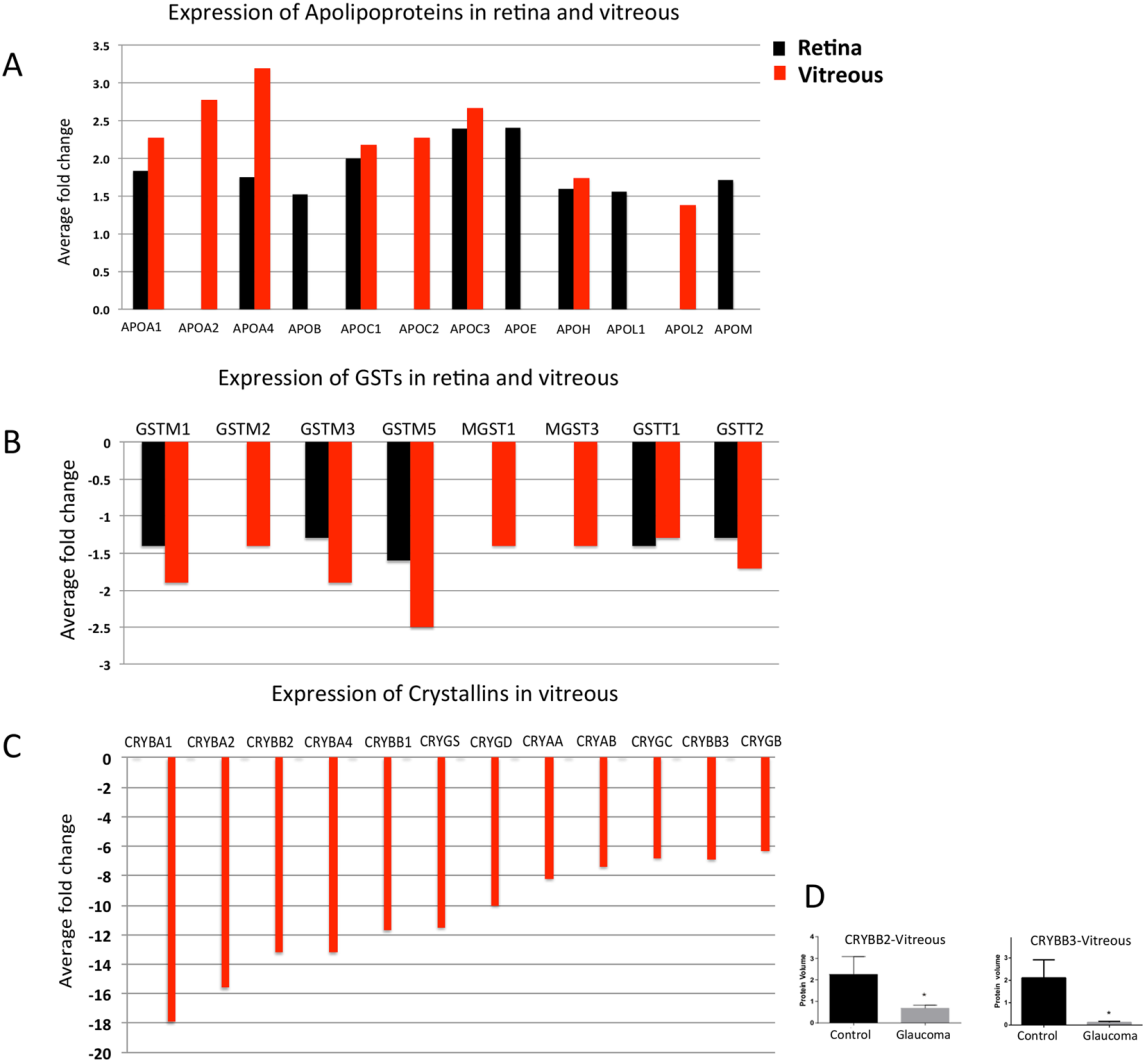
Upregulation of Apolipoproteins and down regulation of Crystallin and GSTs in glaucoma. (**A**) A bar graph representing the expression pattern of 12 differentially expressed Apolipoproteins identified in retina and/or vitreous (p-value ≤ 0.05 and ≥ 1.3-fold change, n = 10). (**B**) A bar graph representing the relative abundance of eight differentially expressed GSTs identified in retina and/or vitreous (p-value ≤ 0.05 and ≤0.77-fold change, n = 10). (**C**) A bar graph representing the relative abundance of 12 differentially expressed crystallin proteins identified in vitreous (p-value ≤ 0.05 and ≤ 0.77-fold change, n = 10). (**D**) Western blotting analysis for measuring the relative protein expression level of CRYBB2 and CRYBB3 in vitreous (n = 10) of glaucoma and control samples. GAPDH was used as the loading control. The bar graphs indicate average densitometry measurements (ImageJ software) (n = 10, average ± SD, *p-value < 0.05). Black bars represent control, while the light grey bars represent glaucoma.

Defects in cholesterol transport and metabolism have been reported in several neurodegenerative diseases^35^ including glaucoma^36^, AD^37–39^, Huntington’s^40,41^, and Parkinson’s disease^42^. Apolipoproteins are lipid transport proteins in the CNS and other tissues^39^. Recent genetic studies highlighted the importance of apolipoprotein gene polymorphisms as key elements and risk factors for a number of neurological diseases including AD^43,44^. This study identified 12 members of apolipoproteins as upregulated in glaucoma compared to samples from control subjects. Of these, five members (APOA1, APOA4, APOC1, APOC3, APOH) were identified in both vitreous and retinal tissues, however a greater abundance related differences were observed in the vitreous. Four other members were specific to the retina (APOB, APOE, APOL1, APOM) and three were detected uniquely in the vitreous (APOA2, APOL2 and APOC2). Interestingly, of the 12 identified apolipoproteins, eight have previously been reported to be associated with neuropathological progression in AD^45–50^ (Fig. 7A).

### Reduced expression of glutathione S-transferase proteins

This study identified three distinct groups of Glutathione-S-Transferases (GSTs): GSTM1, GSTM2, GSTM3 and GSTM5 from the mu family, GSTT1 and GSTT2 from the theta family, and two microsomal GSTs, MGST1 and MGST2. Interestingly, all eight GSTs were identified as down-regulated in glaucoma eyes compared to the control samples (Fig. 7B). Five of these GSTs were quantified in both retina and vitreous, with the extent of regulation consistently greater in the vitreous, while the other three (MGST1, MGST3 and GSTM2) were distinctly affected in the vitreous (Fig. 7B).

### Up-regulation of proteins associated with RNA processing

Defects in RNA splicing occur in several age related neurodegenerative conditions and are reported previously in glaucoma^51^ and AD^52,53^. Accordingly, we identified 29 protein associated with mRNA processing as significantly up regulated in vitreous along with SRSF9 and HNRNPM proteins, which were upregulated in both the retina and vitreous. From this network, 8 members of the serine/arginine-rich splicing factors (SR proteins) were found, which are involved in alternative mRNA splicing *via* the spliceosome, including; SRSF1, SRSF2, SRSF3, SRSF4, SRSF7, SRSF9, SRSF10 and SRSF11. Additionally, 5 members of hnRNPs complexes (Heterogeneous nuclear ribonucleoproteins) HNRNPC, HNRNPH3, HNRNPF, HNRNPM, HNRNPU and 7 members of small nuclear ribonucleoproteins (SNRA, SNRPD2, SNRPD3, SNRPE, SNRP70 and EFTUD2) were upregulated exclusively in the vitreous tissue (Supplementary Figure S3 and Supplementary data S2).

### Down regulation of Crystallins

Crystallins are known as dominant structural proteins of the lens and are classified into 3 main families, alpha, beta and gamma^54^. Due to their significant homology and characteristic similarities with heat shock proteins and chaperones, they are known to play a role in enhancing cellular resistance to ageing induced apoptotic cell death. We identified 11 members of crystallin family as significantly down-regulated exclusively in the vitreous samples. Crystallins were the most differentially expressed proteins in our dataset (up to 18-fold down regulation in glaucoma condition compared to control). The Reactome pathway analysis revealed that crystallins are clustered with proteins associated with cholesterol transport and apoptosis (Supplementary Figure 2). Of these 12 proteins, 7 belonged to the beta (CRYBA1, CRYBA2, CRYBB2, CRYBA4, CRYBB1, and CRYBB3), 2 to alpha (CRYAA, CRYAB) and 3 to the gamma family (CRYGB, CRYGS, and CRYGD). The protein expression of CRYBB3 and CRYBB2 were validated using western blotting approach (Fig. 7C and D).

### Confirmation of proteomics data (selected AD associated markers) using an ECL assay

**Figure 8:**
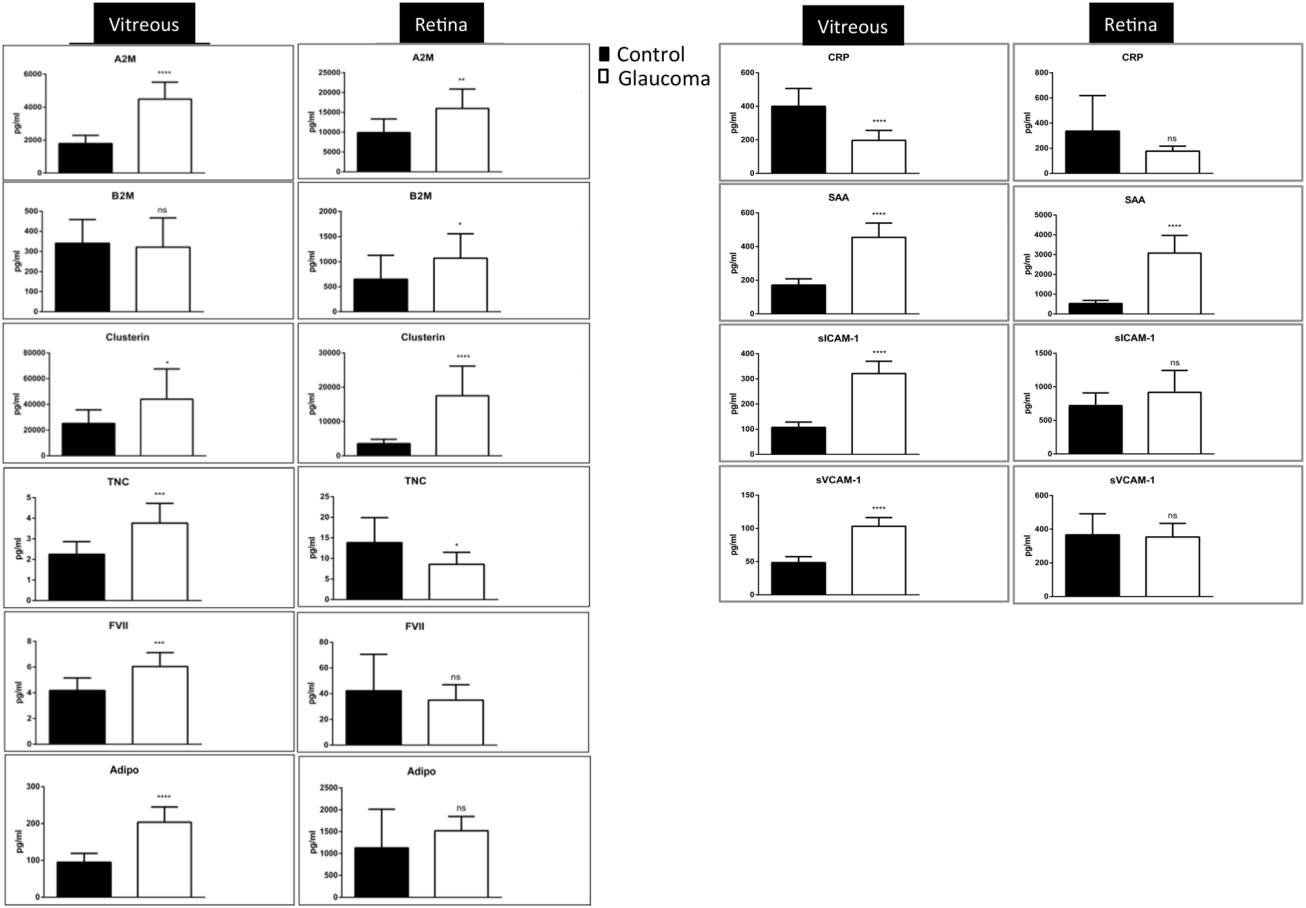
ECL analysis of AD associated markers. ECL analysis of selected proteins (SAA, CRP, sVCAM1, sICAM1, A2M, B2M, FVII, TNC), which are known as AD biomarkers in the literature and also identified in the proteomics data of this study. All samples (n = 10 control and n = 10 glaucoma) were assayed in duplicate via a multiplex biomarker assay platform using ECL on the SECTOR Imager 2400A from Meso Scale Discovery.

To support our quantitative MS results and provide further evidence for the molecular association of glaucoma with other neurodegenerative diseases, specifically AD, we selectively examined expression changes of proteins that are widely reported to be affected in AD, using a quantitative ECL based assay. ECL assay was carried out on ten known AD biomarkers (A2M, B2M, Clusterin, TNC, FVII, Adiponectin, CRP, SAA, ICAM-1 and VCAM-1), which were identified in our proteomics datasets. The association of these markers with AD is extensively reported in the literature^55–60^. The up regulation of A2M, Clusterin, SAA, ICAM-1 VCAM-1 and down regulation of TNC in both tissues was detected, consistent with the proteomics experiments. B2M remained unchanged in vitreous and was up regulated in the retina, while MS analysis showed that both of these markers were up regulated. Similarly, TNC and FVII were up regulated in vitreous and down regulated or unchanged in retina using ECL, but remained unchanged or were not detected in either tissue using proteomics (Fig. 8). This may be attributed to differences in the capture affinity of the detection antibodies used in ECL, potential for cross-reactivity, buffers and other reagents that are used in these assays. Together both these approaches demonstrate that six of these ten markers were similarly affected in the disease condition.

## Discussion

This study used a combination of multiplexed mass spectrometry-based proteomics (TMT labelling), functional protein network and pathways analyses, ECL analysis, and western blotting to delineate global and specific proteomics changes in human retinal and vitreous glaucoma tissues. Only a limited number of proteomics studies have been performed on human glaucoma tissues^61,62^. For instance, Funke *et al*.^61^ has previously reported identifying 600 proteins from human retinal tissues, of which about 10% showed proteome alterations in the glaucomatous tissues. In the current study, our approach analysed both retinal and vitreous tissue proteomes to a significant depth of about 5000 proteins, providing the first comprehensive elucidation of proteome changes in the these tissues from glaucoma patients. We report differential regulation of 122 proteins in retinal and vitreous tissues, which overlap with functional networks associated with neurodegenerative conditions of CNS with a particular focus on AD pathology. We believe that the elucidation of the complex proteome alterations occurring in the vitreous and retinas of glaucoma subjects will serve as a key source for the identification of glaucoma specific biological targets for diagnostic biomarkers, disease prevention, and therapeutic strategies. Our in-depth computational analysis highlights several unique biochemical pathways that are also known to be impaired in AD conditions. The proteomics overlap highlighting similarities and differences between glaucoma and AD will further help to elucidate converging pathological mechanisms underlying AD and various other neurodegenerative disorders.

More specifically, our data analysis revealed that a significant proportion of annotated proteins belonged to categories related to neuronal components and neurological disorders. Pathway enrichment analysis followed by examining functional networks supported the notion that pathways linked to cellular energy regulation, complement pathway activation, antioxidant defence mechanisms and acute phase response signalling were predominantly affected. Our findings also implicate impairment in mitochondrial machinery, cholesterol metabolism, inflammatory responses, regulation of cytoskeletal proteins, receptor mediated endocytosis and retinoid nuclear receptor alterations in glaucoma conditions. Interestingly, we observed significant downregulation of several crystallin proteins in the vitreous of glaucoma subjects. Crystallins are a class of heat shock protein which are suggested to protect neurons by suppressing signals linked to stress mediated apoptosis; therefore, reduced crystallin levels in glaucoma vitreous could make the retinal neurons more vulnerable to cytotoxic injury and cell death. Downregulation of various crystallin proteins and mRNA has previously been reported in a rat model of experimental glaucoma^63,64^.

Several *in vivo* and *in vitro* studies have demonstrated the potential neuroprotective role of crystallins (β-crystallin B2) in survival of injured retinal ganglion cells in rat^65^ and axonal regeneration in the injured optic nerve and photo receptors in animal experimental model^66^. Interestingly the most recent retinal proteomics study of the experimental glaucoma model^64^ reported down regulation of several crystalline proteins 3 weeks after IOP elevation, however, their expression trend was reversed after the 7^th^ week. Furthermore the authors showed that an intravitreal injection of β-crystallin B2 during the IOP elevation is responsible for increasing the retinal ganglion cell survival and protecting against the loss of the retinal nerve fiber layer and optic nerve injury^64^.

Conversely, while AD brains show increased αB-crystallin expression, changes in retinal expression of various crystallins in AD remains unclear^67^.

Proteome characterization of the retina and vitreous tissues, which are primarily associated with glaucoma disease process, represent the first step towards enhancing our molecular understanding of this complex neurodegenerative disorder and explain the development of specific features of the disease. Functional network analysis of the differentially regulated proteins in glaucoma may hold the key to pathognomonic processes associated with other CNS neurodegenerative disorders that have increasingly been shown to affect molecular, anatomical and functional networks in the retina. We also validated our proteomics findings using highly sensitive electro-chemiluminescence assay on MSD platform^58^ to evaluate protein expression of selected AD candidate markers. Consistent with mass spectrometry findings, we observed that A2M, B2M, Clusterin, TNC, FVII, Adiponectin, CRP, SAA, ICAM-1 and VCAM-1 were differentially affected in the retinas and vitreous of glaucoma subjects. Importantly, of these A2M, B2M, Clusterin, TNC, FVII, Adiponectin represent metabolic markers that are modulated in AD^58^. Altered expression of AD associated vascular markers like CRP, SAA, ICAM-1 and VCAM-1 also correlated well with our proteomics analysis. These observations support the hypothesis that glaucoma is associated with both neuronal and vascular stress.

The biochemical interactions identified in several signalling networks elucidated in this study could lead to neurodegeneration through cell apoptosis. The impact of altered metabolic and biochemical function on complex biomolecules can lead to conformational alterations and covalent modifications. It is difficult to determine whether proteome alterations in glaucoma are a consequence or cause of the disease. Our analysis highlighted a significant decrease in proteins associated with regulating glutathione metabolism that directly modulates oxidative stress response, which may reflect an attempt by the retinal and vitreous tissues to neutralize the increased oxidative stress associated with glaucoma pathogenesis. Similarly, we observed a down regulation of proteins involved in mitochondrial function and ATP generation, which may suggest an inability of the retinal tissue to meet the energy requirements leading to shifting of equilibrium to pro-apoptotic pathways. Increased expression of some of the components of complex 1 in vitreous may represent a compensatory effect or efflux of these proteins from dying retinal cells.

Apart from oxidative stress, our study suggests complement cascade involvement in glaucoma. The complement pathway not only plays a critical role in the inflammatory response, but is also involved in synaptic development and pruning. Defective synaptic elimination has been associated with limited refinement of the retinogeniculate pathway. Preferential over-expression of several components of the complement pathway were identified in glaucoma samples. Our study provides a highly inclusive data set regarding alterations in complement proteins. Together with previous observations, our findings corroborate the reported upregulation of C1q complement protein in glaucoma^68^. C1q along with synaptic markers such as PSD95 has been implicated in early glaucoma pathogenesis, suggesting complement activation in synaptic regulation in glaucoma. Increased C1q expression was also reported in neurons in AD animal models^69^. Differential effects on complement pathways is known in other neurodegenerative disorders and amyloid β accumulation in particular is an activator of complement pathway^70^.

Aβ deposits are well known to accumulate in the retinas in both AD and glaucoma conditions^10,71^. Similarly, plaque formation in AD is influenced by cholesterol, chronic oxidative stress, complement activation and neuroinflammation. The inflammatory responses, such as the activation of complement pathways play a fundamental role in synaptic development and pruning, cell death and ultimately in pathophysiology of numerous neurodegenerative diseases, including glaucoma^72,73^. The complement system is activated by three separate pathways; the classical, alternative and lectin^74^. However, among the three pathways, the classical pathway has been shown to be predominantly affected in both AD^33^ and glaucoma^30^. The classical complement pathway is comprised of more than 20 proteins, comprising several serine proteinases linked as an amplifying cascade. There is evidence for the bidirectional cross-talk between the complement and coagulation cascades^75,76^. Since some complement proteins act as substrates for coagulation factors, enhanced levels of coagulation cascade proteins could potentially promote the complement cascade activation. Alternatively, complement activation might also lead to coagulation and fibrin deposition^77^.

We identified for the first time a direct link of apolipoprotein expression in glaucoma. Our study found significant upregulation of various apolipoproteins in the retinas and vitreous of glaucoma subjects. Apo A-I plays a crucial role in cholesterol transport and metabolism and we observed increased Apo A-I concentrations in glaucoma samples. The regulatory effects of Apo-A1 on reverse cholesterol transport pathway have also been suggested in AD. ApoE4 changes were primarily localised to the retina and no overexpression of ApoE4 was identified in the vitreous. Importantly the *APOE* allele variant is a significant risk factor for sporadic type of late onset AD^47^. The *APOE* allele is involved in moderating Aβ proteolysis and clearance, and is associated with both glaucoma and AD effects on the retina^6,10^. This study establishes the changes in expression of various members of the apolipoprotein family, indicating significant alterations in apolipoprotein metabolism. It is thus highly likely that apolipoproteins may form potential biomarkers to predict the effects of glaucoma in the future.

Increased ROS production underlies oxidative damage to macromolecules in the cells, including lipids, proteins and nucleic acids. These changes have been directly associated with aging and neurodegeneration^27^. Association between the production of oxidant/free radical species, dysfunction of the antioxidant system, and the onset and progression of neurodegenerative diseases has been increasingly well established^78,79^. For instance, glutathione (GSH), a thiol-containing tripeptide, and GSH-related enzymes (GSTs) are important endogenous antioxidant components that are involved in mediating protection of cells against oxidative stress by neutralising highly reactive free radical species^80^. GSTs catalyse the conjugation of reduced GSH to xenobiotic compounds, which are less reactive and more soluble, and hence facilitate clearance from the body. GSH synthetases regulate the GSH system in both cytoplasm and endoplasmic reticulum and dysregulation of the system is linked with neurodegeneration^81^. GST has also been described as a glaucoma associated stress marker, and increased serum GST immunoreactivity was reported in glaucoma patients. Furthermore, polymorphisms of selected GSTs such as GSTM1 and GSTT1 were shown to be associated with the onset and development of glaucoma^82,83^, cataract^84,85^, PD^86,87^ and AD^88^.

A limitation of our approach is that we analysed whole retinal tissues, and thus cannot distinguish the individual contribution of distinct cell types that comprise the retina. This may lead to changes in proteins in the inner retinal region, which are particularly affected in glaucoma to be masked, or lead to significantly diminished fold alterations. Future studies may use techniques like laser dissection microscopy in the unfixed tissues to identify region and layer specific proteomics changes in the retina. Immunostaining of the retinal sections with selected markers will further help to elucidate the layer/region specific protein expression differences in the retina under glaucoma conditions. The protein expression changes in vitreous may similarly be caused by efflux of proteins from retinas or other ocular tissues and may not reflect *bona fide* vitreous proteome alterations. Further, although a great deal of reproducibility was observed between the samples suggesting differential regulation of proteins in the disease state, detailed information from the post-mortem donor samples including the drug treatments etc. will help for in-depth analysis of the data. Nevertheless, this study enables us to understand the complex nature of disease-associated pathology in retinal and vitreous tissues although it does not suggest whether it is a cause or effect of the chronic disease condition. Furthermore, the downregulation of several proteins in the retina may be caused by loss of neuronal cells in glaucoma leading to reduced protein quantity from particular retinal cells. This may reflect reduced expression at the tissue level and not at individual neuron levels.

Overall, very few studies have tried to substantiate the molecular similarities and differences between open angle glaucoma and more generalised CNS neurodegenerative disorders such as AD. The comprehensive profile of the eye proteome changes identified in this study provides novel insights into the pathobiology of glaucoma. Similarities and differences between various proteins in glaucoma and other neurodegenerative conditions may lead to the development of novel drug targets and disease specific biomarker identification. Our results provide a significant advancement towards a better understanding of the mechanistic basis of glaucoma and its association with other neurodegenerative diseases.

## Electronic supplementary material

Supplementary Figures

Supplementary Dataset S1

Supplementary Dataset S2
