# Supplementary material for: Age-related neurodegenerative disease associated pathways identified in retinal and vitreous proteome from human glaucoma eyes
Source: Sci Rep. 2017 Oct 4;7:12685. doi: 10.1038/s41598-017-12858-7 (PMC5627288; doi:10.1038/s41598-017-12858-7)
Supplement: Supplementary file 1 — Supplementary Figures [file 41598_2017_12858_MOESM1_ESM.pdf]

Age-related neurodegenerative disease associated pathways identified in retinal and vitreous proteome from human glaucoma eyes

Mehdi Mirzaei[1, 9★], Veer B Gupta[2], Joel M Chick[3], Todd M Greco[4], Yunqi Wu[1], Nitin Chitranshi[5], Roshana Vander Wall[5], Eugene Hone[2], Liting Deng[1], Yogita Dheer[5], Mojdeh Abbasi[5], Mahdie Rezaeian[5], Nady Braidy[6], Yuyi You[5, 7], Ghasem Hosseini Salekdeh[8], Paul A Haynes[1], Mark P Molloy[1, 9], Ralph Martins[2, 5], Ileana M Cristea[4], Steven P Gygi[3], Stuart L Graham[5, 7] and Vivek K Gupta[5]

[1] Department of Chemistry and Biomolecular Sciences, Macquarie University, NSW, Australia

[2]School of Medical Sciences, Edith Cowan University, Joondalup, WA, Australia

[3]Department of Cell Biology, Harvard Medical School, Boston, Massachusetts, USA

[4]Department of Molecular Biology, Princeton University, Princeton, New Jersey, USA

[5]Faculty of Medicine and Health Sciences, Macquarie University, NSW, Australia

[6]Centre for Healthy Brain Ageing, School of Psychiatry, University of New South Wales, Sydney, Australia

[7]Save Sight Institute, Sydney University, NSW, Australia.

[8]Department of Molecular Systems Biology, Cell Science Research Center, Royan Institute for Stem Cell Biology and Technology, ACECR, Tehran, Iran

[9]Australian Proteome Analysis Facility, Macquarie University, NSW, Australia

★Corresponding author:

Mehdi Mirzaei

Department of Chemistry and Biomolecular Sciences, Macquarie University, NSW, Australia

Australian Proteome Analysis Facility, Macquarie University, NSW, Australia

Tel: +61 98508284

Email: mehdi.mirzaei@mq.edu.au

Supplementary information

Supplementary dataset 1- The list of proteins identified in the retina samples, pairwise comparison test of glaucoma vs. control

The combined set of proteins identified from two separate TMT experiments performed on retinal samples in glaucoma and control conditions, as well as the list of differentially expressed proteins obtained from a t-test comparison test between glaucoma and control.

Supplementary dataset 2- The list of proteins identified in the vitreous samples- pairwise comparison test of glaucoma vs. control

The combined set of proteins identified from two separate TMT experiments performed on vitreous samples in glaucoma and control conditions as well as the list of differentially expressed proteins obtained from a t-test comparison test between glaucoma and control.

Supplementary Figure S1- Sample densities and box plots of TMT experiments

A series of descriptive statistical analyses were performed to confirm the reproducibility of the data. The overall distribution of median-normalized and log-transformed protein abundances were visualised in density (A) and box plots (B). Within each tissue (retina and vitreous), the boxplots showed similar median and 95% confidence intervals. Moreover, comparison of density plots for each individual biological sample showed similar and highly overlapping patterns, with no major asymmetric bias, satisfying the normality assumption for further analysis. Overall, these statistical metrics confirm the similarity in overall protein abundance distributions of individual biological replicates, and demonstrate the reproducibility of measurements within sample groups.

Supplementary Figure S2-Vitreous functional protein network clustered by Reactome

Vitreous Reactome functional interaction networks analyzed by the Reactome FI Cytoscape plugin. Of 1153 vitreous differentially expressed proteins, 568 proteins were had at least one other known functional connection. Network nodes are labeled with gene symbols. The Reactome plugin was used to assign functional clusters, which were color-coded and labeled with representative broad functions. Large number of network clusters was identified representing wide array of cellular pathways in the vitreous.

Supplementary Figure S3-Abundance-coded vitreous reactome functional network

The Reactome plugin was used to assign functional clusters and color-coded (based on their abundance) and labeled with representative broad functions. Yellow represent up-regulation and blue denote upregulation.

Figure S1

Retina

Vitreous

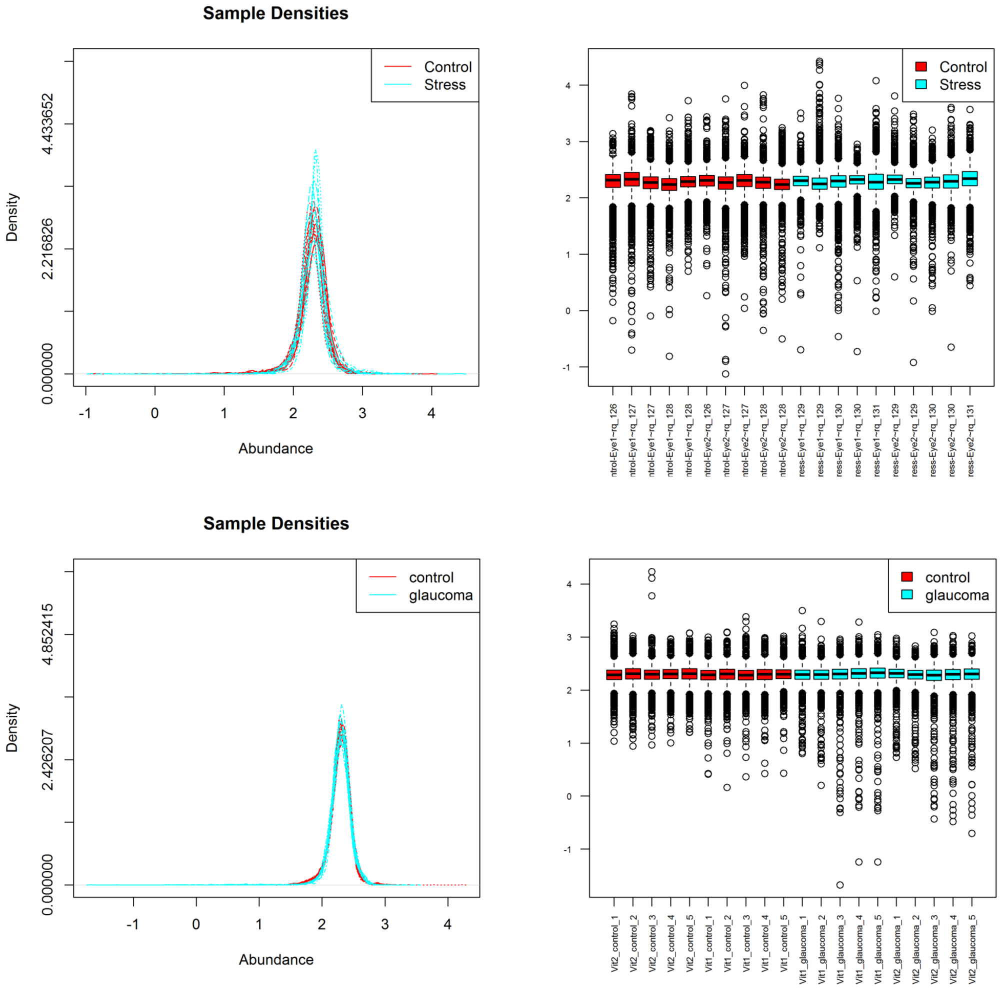

Figure S2

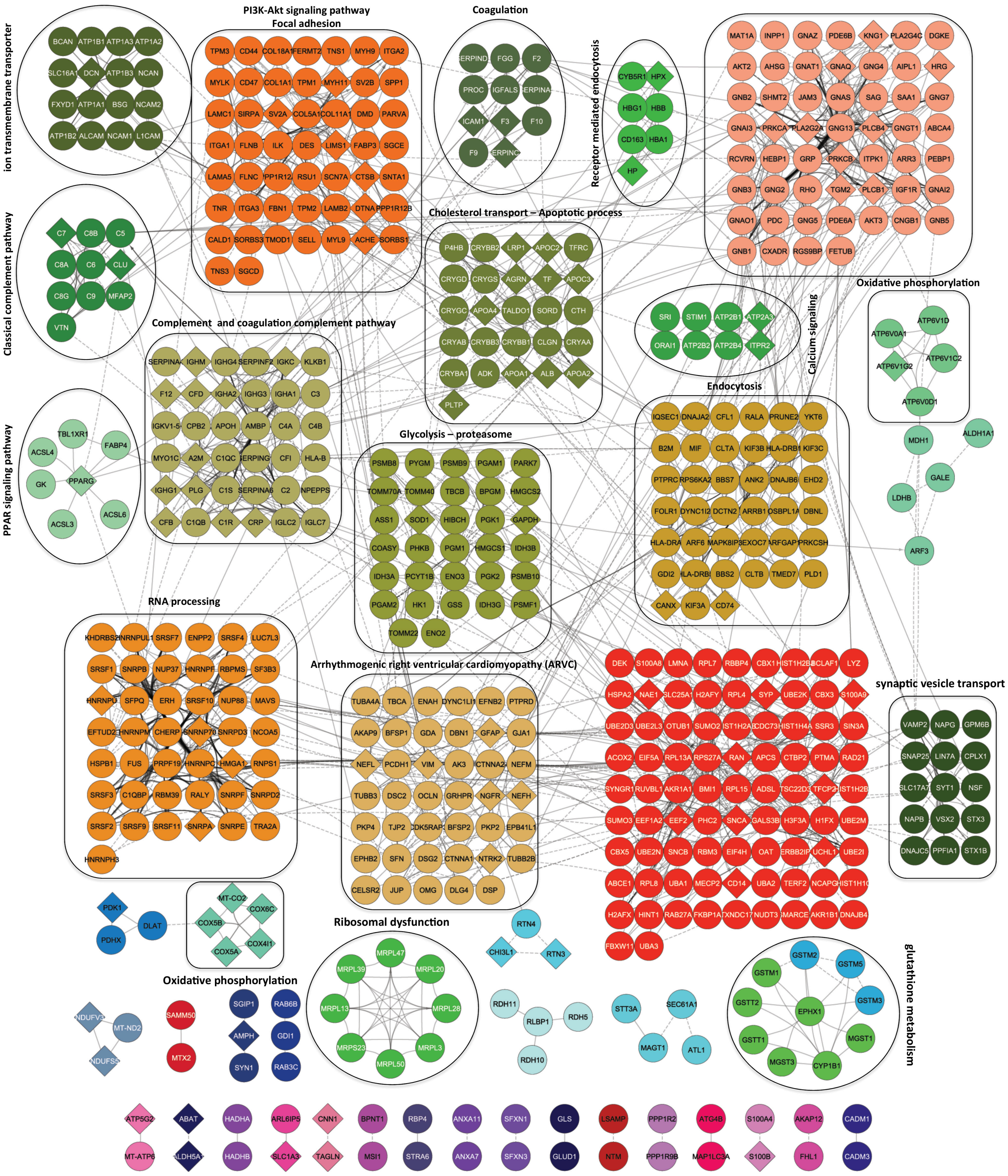
ion transmembrane transporter
PI3K-Akt signaling pathway
Focal adhesion
Coagulation
Receptor mediated endocytosis
Classical complement pathway
Cholesterol transport – Apoptotic process
Complement and coagulation complement pathway
Oxidative phosphorylation
Calcium signaling
Endocytosis
PPAR signaling pathway
Glycolysis – proteasome
RNA processing
Arrhythmogenic right ventricular cardiomyopathy (ARVC)
synaptic vesicle transport
Ribosomal dysfunction
Oxidative phosphorylation
glutathione metabolism

Figure S3

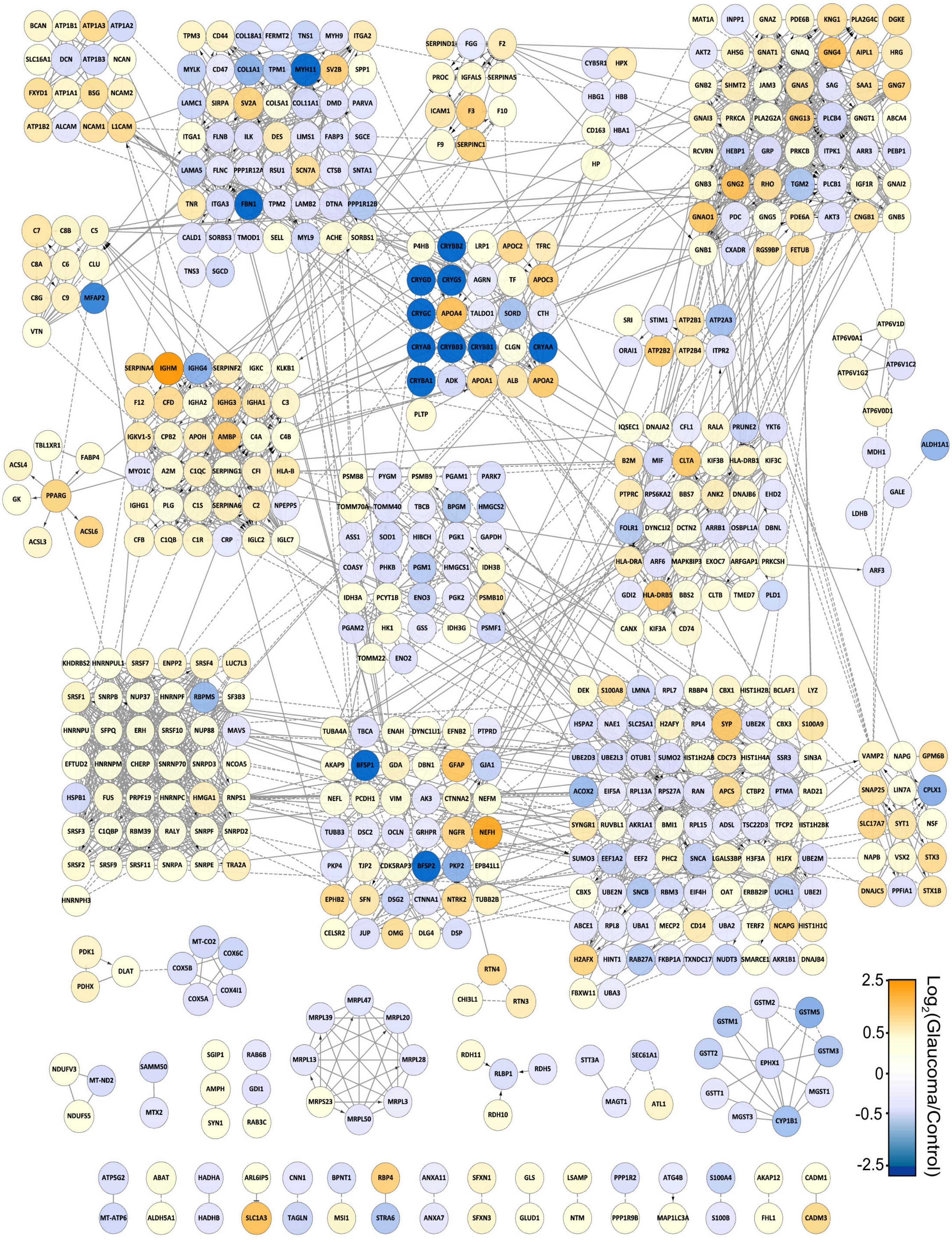
BCAN ATP1B1 ATP1A3 ATP1A2
SLC16A1 DCN ATP1B3 NCAN
FXYD1 ATP1A1 BSG NCAM2
ATP1B2 ALCAM NCAM1 L1CAM
TPM3 CD44 COL18A1 FERMT2 TNS1 MYH9 ITGA2
MYLK CD47 COL1A1 TPM1 MYH11 SV2B SPP1
LAMC1 SIRPA SV2A COL5A1 COL11A1 DMD PARVA
ITGA1 FLNB ILK DES LIMS1 FABP3 SGCE
LAMA5 FLNC PPP1R12A RSU1 SCN7A CTSB SNTA1
TNR ITGA3 FBN1 TPM2 LAMB2 DTNA PPP1R12B
CALD1 SORBS3 TMOD1 SELL MYL9 ACHE SORBS1
TNS3 SGCD
SERPIND1 FGG F2
PROC IGFALS SERPINA5
ICAM1 F3 F10
F9 SERPINC1
CYB5R1 HPX
HBG1 HBB
CD163 HBA1
HP
MAT1A INPP1 GNAZ PDE6B KNG1 PLA2G4C DGKE
AKT2 AHSG GNAT1 GNAQ GNG4 AIPL1 HRG
GNB2 SHMT2 JAM3 GNAS SAG SAA1 GNG7
GNAI3 PRKCA PLA2G2A GNG13 PLCB4 GNGT1 ABCA4
RCVRN HEBP1 GRP PRKCB ITPK1 ARR3 PEBP1
GNB3 GNG2 RHO TGM2 PLCB1 IGF1R GNAI2
GNAO1 PDC GNG5 PDE6A AKT3 CNGB1 GNB5
GNB1 CXADR RGS9BP FETUB
C7 C8B C5
C8A C6 CLU
C8G C9 MFAP2
VTN
P4HB CRYBB2 LRP1 APOC2 TFRC
CRYGD CRYGS AGRN TF APOC3
CRYGC APOA4 TALDO1 SORD CTH
CRYAB CRYBB3 CRYBB1 CLGN CRYAA
CRYBA1 ADK APOA1 ALB APOA2
PLTP
SRI STIM1 ATP2B1 ATP2A3
ORAI1 ATP2B2 ATP2B4 ITPR2
ATP6V1D ATP6V0A1 ATP6V1C2 ATP6V1G2 ATP6V0D1
SERPINA4 IGHM IGHG4 SERPINF2 IGKC KLKB1
F12 CFD IGHA2 IGHG3 IGHA1 C3
IGKV1-5 CPB2 APOH AMBP C4A C4B
MYO1C A2M C1QC SERPING1 CFI HLA-B
IGHG1 PLG C1S SERPINA6 C2 NPEPPS
CFB C1QB C1R CRP IGLC2 IGLC7
TBL1XR1 FABP4 ACSL4 GK PPARG ACSL6 ACSL3
PSMB8 PYGM PSMB9 PGAM1 PARK7
TOMM70A TOMM40 TBCB BPGM HMGCS2
ASS1 SOD1 HIBCH PGK1 GAPDH
COASY PHKB PGM1 HMGCS1 IDH3B
IDH3A PCYT1B ENO3 PGK2 PSMB10
PGAM2 HK1 GSS IDH3G PSMF1
TOMM22 ENO2
IQSEC1 DNAJA2 CFL1 RALA PRUNE2 YKT6
B2M MIF CLTA KIF3B HLA-DRB1 KIF3C
PTPRC RPS6KA2 BBS7 ANK2 DNAJB6 EHD2
FOLR1 DYNC1I2 DCTN2 ARRB1 OSBPL1A DBNL
HLA-DRA ARF6 MAPK8IP3 EXOC7 ARFGAP1 PRKCSH
GDI2 HLA-DRB5 BBS2 CLTB TMED7 PLD1
CANX KIF3A CD74
ALDH1A1 MDH1 GALE LDHB ARF3
KHDRBS2 HNRNPUL1 SRSF7 ENPP2 SRSF4 LUC7L3
SRSF1 SNRPB NUP37 HNRNPF RBPMS SF3B3
HNRNPU SFPQ ERH SRSF10 NUP88 MAVS
EFTUD2 HNRNPM CHERP SNRNP70 SNRPD3 NCOA5
HSPB1 FUS PRPF19 HNRNPC HMGA1 RNPS1
SRSF3 C1QBP RBM39 RALY SNRPF SNRPD2
SRSF2 SRSF9 SRSF11 SNRPA SNRPE TRA2A
HNRNPH3
TUBA4A TBCA ENAH DYNC1LI1 EFNB2 PTPRD
AKAP9 BFSP1 GDA DBN1 GFAP GJA1
NEFL PCDH1 VIM AK3 CTNNA2 NEFM
TUBB3 DSC2 OCLN GRHPR NGFR NEFH
PKP4 TJP2 CDK5RAP3 BFSP2 PKP2 EPB41L1
EPHB2 SFN DSG2 CTNNA1 NTRK2 TUBB2B
CELSR2 JUP OMG DLG4 DSP
DEK S100A8 LMNA RPL7 RBBP4 CBX1 HIST1H2B BCLAF1 LYZ
HSPA2 NAE1 SLC25A1 H2AFY RPL4 SYP UBE2K CBX3 S100A9
UBE2D3 UBE2L3 OTUB1 SUMO2 HIST1H2AB CDC73 HIST1H4A SSR3 SIN3A
ACOX2 EIF5A RPL13A RPS27A RAN APCS CTBP2 PTMA RAD21
SYNGR1 RUVBL1 AKR1A1 BMI1 RPL15 ADSL TSC22D3 TFCP2 HIST1H2BK
SUMO3 EEF1A2 EEF2 PHC2 SNCA LGALS3BP H3F3A H1FX UBE2M
CBX5 UBE2N SNCB RBM3 EIF4H OAT ERBB2IP UCHL1 UBE2I
ABCE1 RPL8 UBA1 MECP2 CD14 UBA2 TERF2 NCAPG HIST1H1C
H2AFX HINT1 RAB27A FKBP1A TXNDC17 NUDT3 SMARCE1 AKR1B1 DNAJB4
FBXW11 UBA3
VAMP2 NAPG GPM6B
SNAP25 LIN7A CPLX1
SLC17A7 SYT1 NSF
NAPB VSX2 STX3
DNAJC5 PPFIA1 STX1B
PDK1 PDHX DLAT
MT-CO2 COX6C COX5B COX4I1 COX5A
RTN4 CHI3L1 RTN3
MRPL47 MRPL39 MRPL20 MRPL13 MRPL28 MRPS23 MRPL50 MRPL3
GSTM2 GSTM1 GSTM5 GSTT2 EPHX1 GSTM3 GSTT1 MGST1 MGST3 CYP1B1
NDUFV3 MT-ND2 NDUFS5
SAMM50 MTX2
SGIP1 AMPH SYN1
RAB6B GDI1 RAB3C
RDH11 RLBP1 RDH5 RDH10
STT3A SEC61A1 MAGT1 ATL1
ATP5G2 MT-ATP6
ABAT ALDH5A1
HADHA HADHB
ARL6IP5 SLC1A3
CNN1 TAGLN
BPNT1 MSI1
RBP4 STRA6
ANXA11 ANXA7
SFXN1 SFXN3
GLS GLUD1
LSAMP NTM
PPP1R2 PPP1R9B
ATG4B MAP1LC3A
S100A4 S100B
AKAP12 FHL1
CADM1 CADM3
2.5
0.5
0
-0.5
-2.5
Log2(Glaucoma/Control)
